# Reminiscences of Castration

**Published:** 1895-08

**Authors:** M. J. Treacy

**Affiliations:** Fort Meade, S. D.


### REMINISCENCES OF CASTRATION.

BY M. J. TREACY, M.R.C.V.S.,
FORT MEADE, S. D.

Whilst altering a stallion this spring a one-session student remarked to me, " I cannot understand all the fuss they make over the so-called bad results of castration. I have never seen any so far after watching it closely now for several years."

Now, I do not propose, were I capable of so doing, to write a learned treatise on the above subject, but will endeavor to submit my practical experience for nearly twenty-five years.

The above-quoted remark struck me very forcibly as being applicable to myself years ago, for the first fifteen years of my professional career I never to my knowlege had a bad result from castration under those lucky circumstances (too lucky to last, as I subsequently found out). Was it any wonder for me to think as my junior *confrère* did? In that time I altered hundreds of horses successfully by every known method—torsion; clamps; caustic, and plain, covered and uncovered actual cautery; ligature; pulping the cords with a blunt hammer, without any incision. About 1886 I adopted the ècraseur; after this I commenced having a very large and disheartening percentage of schirrous cords, all of them acute, and occurring within one or two weeks after the operation. It was simply astonishing the magnitude they would assume in a few days' time. Seven days after castration I removed from one stallion two cords (schirrous) each over three inches in circumference and weighing together not less than two pounds. For several years (about four or five) this bad luck continued, so much so that I contemplated seriously giving up the operation, whilst surrounding me on all sides were ignorant quacks with filthy hands, tobacco-stained knives, etc. (one of them always filling the scrotum after the operation with cow or horse excreta), castrating horses with apparent success. Was I idle during those years in trying to enlighten myself as to the cause of my failure? No, I was not. I worked hard, "boned" up all authors on this subject—German, English, French—that I could get hold of, and as I read some assigned cause for my failure from a standard authority I either purchased old stallions cheap or else operated on a subject the property of some confiding friend. Needless to say that my instrument and hands, etc., were always aseptic, so far as I could make them, so the author ascribing the bad results to septic poisoning did not suit me; in fact, I try now to promote suppuration instead of endeavoring to heal the scrotal wounds by first intention, as recommended by many authors.

*Leaving the Cord Too Long.* I have for one season taken the cords off as high as I could reach them, still bad results; and taken them off every length, no better luck. I have frequently seen cords hanging one inch out of the scrotum with no ill results.

*Large Incisions.* Always made them.

*Dragging the Cord.* I have seen cord stretched by an Indian medicine man until they gave way hundreds of times and never saw bad results. I do not recommend this practice.

*Catching Cold.* This so-called reason is scarcely in keeping with modern scientific ideas.

*Failure to Sever the Posterior Septum Attaching the Parietal and Visceral Scrotal Layers.* Although I always sever this, I

have seen it neglected in hundreds of cases by successful castrators. One friend of mine, who operates on over two hundred horses of all ages annually and successfully, never pays any attention to this matter.

*Hemorrhage.* But no authority that I could find gives this, and here I found that the chief cause of my troubles lay. Show me one hundred horses that bled freely from their spermatic arteries after castration, and there will be no difficulty in finding fifty cases of schirrous cords. Since I accidentally discovered this I have no more trouble, and how grateful I feel, so much so that I now feel pleasure in castrating a horse of any age, when a few years ago I approached the operation without confidence and with all sorts of miserable anticipations.

Spring of 1891, I think, an aged stallion was brought to me for operation. Observing him to be of a lymphatic temperament, I took the precaution to crush the cords partially with ecraseur before finally crushing them off half an inch lower down. Notwithstanding this, when the horse assumed the erect position he bled profusely from both cords. Now, it is all very well to tell the owner and lookers-on under those circumstances "the bleeding will do him good, etc.," and all the well-known stereotyped phrases usually made use of under those unpleasant surroundings, but this does not prevent the operator from having, as as friend of mine expressed it, "the cold chills running up and down his spine." Luckily bethought me of my lawn hose. A stream from this on to the scrotum acted like magic. I have since tried it with the most gratifying success. (I recommend this procedure to the memory of your readers strongly.) Being dubious of letting the horse go home, a distance of twelve miles, I kept him over night. Next morning I removed from each scrotal sac enough clotted blood to fill a pint measure. Eureka! had the blood not been removed, the lower part of it sealing the mass above from atmospheric influences, it would have been organized into low fibrous tissue, and a continuation of the cords of an enormous size, constituting schirrus, in a few days. I have tested this theory now for several seasons, and to my entire satisfaction. I rarely had a schirrus where there was not hemorrhage from the spermatic arteries, which stopped by reason of the scrotum filling up with coagulated blood. Thinking that the ligature would fulfil my theory, I read up the subject again, and found that Williams's *Surgery* (page 646) states: "The operating by ligature of the artery seems the most surgical and humane, but experience has proved that it is the most unsuccessful of all methods. The late Professor Dick recommended the ligature for a number of years, but toward the end of his life he was forced to acknowledge, and frankly did so, that it was attended with frequent fatal results, the very presence of the ligature seemingly inducing a prejudicial effect, irritating the cord and causing peritonitis or abscesses."

Without for one moment doubting the above or putting my humble opinion against such learned men as Professors Dick or Williams, all the consequences stated above can be produced at will by using a thick ligature.

I have now castrated about thirty aged stallions by first using bichloride freely on the scrotum by means of soap and a brush applied vigorously, making a large incision well back and as near the frenum scroti as possible so as to get drainage, ligaturing the anterior fasciculus only with a *carbolized waxed-silk thread tied as tight as possible* in a surgeon's knot, having a suppurant, as oil of turpentine or carbolic acid and oil, 1 : 8, introduced freely up the scrotum after operation and each day for a week, lots of gentle exercise, and cleanliness. I now approach this operation with confidence and pleasure.

Whilst the ecraseur is, no doubt, a very valuable instrument, there are times when it fails in its special functions as a hemostatic, and right here is where the several dangers arise, as I have endeavored to explain. It is said an animal never dies from hemorrhage after castration. I have seen five such deaths. I do not mean the slow dripping of blood for days where some septic matter has been introduced during the operation. I mean where the hemorrhage was profuse from the operation until death ensued. At college I saw an old stallion and donkey experimented upon this way. Although the hemorrhage was profuse it stopped in both cases spontaneously, and in each, when examined on the dissecting tables, there was an enormous clot filling the scrotum, *which would have become schirrous cords had the animals lived a week or two longer.*

Now, Mr. Editor, I have been a subscriber for your very valuable paper for many years, and it has often struck me what a wonderful record of cases successfully treated appeared therein year by year. Let us hear a little more of the unsuccessful records, for, according to the late Professor Simonds, "There is more information to be gained from one unsuccessful case and an autopsy, than from one hundred successfully treated."

I trust this subject may meet the eyes of some experienced castrators and induce them to give their opinions through your columns, for reference to the standard authorities on the subject of "Etiology of Schirrous Cord" is vague, unsatisfactory, and unpractical, at least I have found it so.

